# Consistency between self-reported disease diagnosis and clinical assessment and under-reporting for chronic conditions: data from a community-based study in Xi’an, China

**DOI:** 10.3389/fpubh.2024.1296939

**Published:** 2024-01-16

**Authors:** Haobiao Liu, Yanru Zhao, Lichun Qiao, Congying Yang, Ying Yang, Tianxiao Zhang, Qian Wu, Jing Han

**Affiliations:** ^1^Department of Epidemiology and Biostatistics, School of Public Health, Xi'an Jiaotong University Health Science Center, Xi'an, Shaanxi, China; ^2^Department of Occupational and Environmental Health, School of Public Health, Xi'an Jiaotong University Health Science Center, Xi'an, Shaanxi, China; ^3^Department of Endocrinology, The First Affiliated Hospital of Xi'an Jiaotong University, Xi'an, Shaanxi, China; ^4^National Anti-Drug Laboratory Shaanxi Regional Center, Xi'an, Shaanxi, China; ^5^Key Laboratory for Disease Prevention and Control and Health Promotion of Shaanxi Province, Xi'an, Shaanxi, China

**Keywords:** chronic condition, self-report, clinical assessment, under-reporting, community-based survey

## Abstract

**Aims:**

The current study aims to investigate the consistency between the surveyees’ self-reported disease diagnosis and clinical assessment of eight major chronic conditions using community-based survey data collected in Xi’an, China in 2017. With a focus on under-reporting patients, we aim to explore its magnitude and associated factors, to provide an important basis for disease surveillance, health assessment and resource allocation, and public health decision-making and services.

**Methods:**

Questionnaires were administered to collect self-reported chronic condition prevalence among the study participants, while physical examinations and laboratory tests were conducted for clinical assessment. For each of the eight chronic conditions, the sensitivity, specificity, under-reporting, over-reporting, and agreement were calculated. Log-binomial regression analysis was employed to identify potential factors that may influence the consistency of chronic condition reporting.

**Results:**

A total of 2,272 participants were included in the analysis. Four out of the eight chronic conditions displayed under-reporting exceeding 50%. The highest under-reporting was observed for goiter [85.93, 95% confidence interval (CI): 85.25–86.62%], hyperuricemia (83.94, 95% CI: 83.22–84.66%), and thyroid nodules (72.89, 95% CI: 72.02–73.76%). Log-binomial regression analysis indicated that senior age and high BMI were potential factors associated with the under-reporting of chronic condition status in the study population.

**Conclusion:**

The self-reported disease diagnosis by respondents and clinical assessment data exhibit significant inconsistency for all eight chronic conditions. Large proportions of patients with multiple chronic conditions were under-reported in Xi’an, China. Combining relevant potential factors, targeted health screenings for high-risk populations might be an effective method for identifying under-reporting patients.

## 1 Introduction

The global burden of chronic diseases is steadily escalating. In 2019, it was estimated that over 7.1 billion people worldwide were afflicted by chronic illnesses (1). Notably, chronic conditions accounted for approximately 90% of mortality and 85% of disability-adjusted life years in China (2). Among the middle-aged and older adults, 61.9% were found to suffer from at least two chronic conditions, significantly amplifying healthcare expenditures and medical resource utilization (3). This burgeoning prevalence of chronic ailments underscores the pressing need for comprehensive strategies to mitigate the associated health and economic repercussions. According to the 10th edition of the International Diabetes Federation (IDF) Diabetes Atlas released in 2021, China has the highest number of adult diabetes patients in the world. It is projected that by 2045, the number of diabetes patients in China will reach 174 million (4).

Accurate estimation of the prevalence of chronic conditions and its risk factors is essential for the development of effective public health policies and health interventions. However, the prevalence assessment presents certain challenges. Currently, the main methods commonly used to assess the prevalence of chronic conditions include (1) using self-reported disease diagnosis from survey responses; (2) employing disease-specific diagnosis codes found in administrative data; and (3) application of objective measurements and biomarkers collection in health examination surveys (5–12). Self-reported disease diagnosis is prevalence data obtained through questionnaire surveys. While this approach offers the benefits of extensive survey coverage and reduced expenses, it is important to note that the observed prevalence rate may be influenced by individual subjective awareness, memory biases, and societal expectations. Furthermore, what’s even more crucial is that self-reported disease-free participants may not necessarily be genuinely free from illness, they may simply lack a confirmed diagnosis. Over the past few decades, a large number of studies have validated the consistency of self-reported disease diagnosis among community residents (13–18). Findings show that there is a discrepancy between patients’ self-reported disease diagnosis and their true medical conditions, and the direction and magnitude of the discrepancy varies by disease type and individual characteristics (13–18). Inconsistency, including both under-reporting (patients reporting themselves as disease-free) and over-reporting (healthy individuals self-reporting having an illness), may be influenced by a variety of factors, including an individual’s socioeconomic status, education level, gender, age, and health literacy.

For certain diseases, such as hypertension and diabetes, patients who are unaware of their medical condition might lead to serious comorbidity. Hardly any intervention measures could be implemented as these patients perceive themselves to be in good health. Many patients only come to understand the true extent of their medical condition when they are brought into the emergency room by an ambulance and miss the best time for diagnosis and treatment of the disease. On the other hand, for conditions like thyroid nodules, the primary concern is the potential for overdiagnosis. Thyroid nodules are commonly found in individuals with normal thyroid function and typically do not cause neither pressure symptoms nor cosmetic problems (19, 20). The overwhelming majority, exceeding 95%, of thyroid nodules are noncancerous (benign) (21). Therefore, correctly identifying patients’ true medical conditions can contribute to disease control and management as well as reduce the potential medical burden caused by overdiagnosis.

The current study aims to investigate the consistency between the surveyees’ self-reported disease diagnosis and clinical assessment of eight major chronic conditions including hyperthyroidism, hypothyroidism, goiter, thyroid nodules, hypertension, diabetes, hyperlipidemia, and hyperuricemia, using community-based survey data of Xi’an, a major city of China. With a focus on under-reporting, we aim to explore its magnitude and associated factors, to provide an important basis for disease surveillance, public health decision-making and services.

## 2 Materials and methods

### 2.1 Study participants

**Figure 1:**
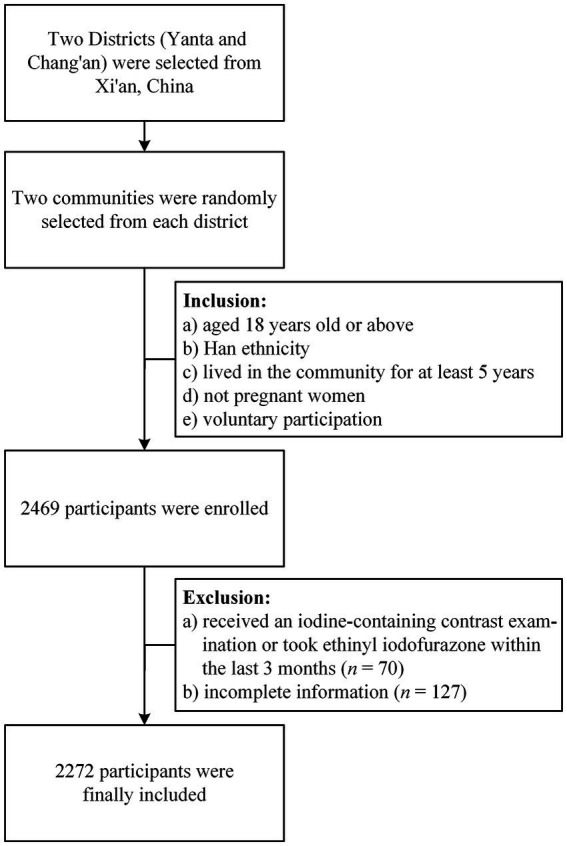
Recruitment process diagram of the study participants.

This study is derived from the Thyroid Disorders, Iodine Status, and Diabetes Epidemiological Survey (TIDE study) conducted in Xi’an, Shaanxi Province in 2017. The TIDE study is led by China Medical University which aimed to obtain information on the iodine status and prevalence of thyroid disorders after the introduction of universal salt iodization in China, as well as the prevalence of diabetes, hypertension, dyslipidemia, and hyperuricemia among adults nationwide. More detailed information can be found in our previous publications (22, 23). To ensure a representative sample of the target population, we employed a multi-stage stratified cluster random sampling method in Xi’an. Specifically, we selected Yanta District and Chang’an District as our primary sampling strata and randomly selected two communities from each district. We limited our sample to individuals who were of Han ethnicity, at least 18 years old, and had resided in the community for a minimum of 5 years. Pregnant women were excluded from the study. Exclusion criteria included recent iodine-containing contrast exams or medication, as well as the requirement for complete information. As a result of these procedures, a total of 2,272 study participants were recruited (Figure 1).

### 2.2 Data collection and measurement

Data collection and measurement processes were conducted by extensively trained technicians. A standardized questionnaire was administered to gather information on demographic and social determinants of health, including residential area, sex, age, ethnicity, education level, occupation, annual family income, iodine nutritional status (such as iodine-containing medication intake and salt consumption), lifestyle behaviors (including smoking status), and past medical history. A total of eight major chronic diseases including hyperthyroidism, hypothyroidism, goiter, thyroid nodules, hypertension, diabetes, hyperlipidemia, and hyperuricemia were investigated in the current study. Participants who responded “yes” to the items “Have you ever been diagnosed with (chronic conditions, mentioned above) by a clinical physician?” were considered as their corresponding self-reported outcomes.

Physical examinations were conducted, during which participants’ height and weight were measured while wearing loose clothing and without shoes and hats. Blood pressure was measured using a high-tech electronic sphygmomanometer (Omron HEM-7430) on the right upper limb after a 10-min rest period. Each blood pressure measurement was performed twice, with a five-minute interval between measurements. Thyroid function was assessed using a uniform portable instrument (LOGIQ 100 PRO, GE, Milwaukee, USA) equipped with 7.5 MHz linear transducers.

All participants were required to fast for at least 8 h overnight, and then their venous blood was collected the following morning for laboratory testing. Several indicators were measured, including fasting blood glucose (FBG), 2-h blood glucose in the oral glucose tolerance test (2 h-OGTT), total cholesterol (TC), triglycerides (TG), low-density lipoprotein cholesterol (LDL-C), and high-density lipoprotein cholesterol (HDL-C), using Myriad kits and biochemical tests. Thyroid-stimulating hormone (TSH) and uric acid levels were measured using a Cobas 601 analyzer (Roche Diagnostics, Switzerland). Additionally, free thyroxine (FT4) levels were assessed when TSH was elevated, and both FT4 and free triiodothyronine (FT3) levels were measured after TSH was lowered.

### 2.3 Clinical and health assessment

Thyroid disorders were defined based on established diagnostic criteria from previous studies (Supplementary Table S1). Hypertension was identified by systolic blood pressure (SBP) ≥140 mmHg and/or diastolic blood pressure (DBP) ≥90 mmHg or prior diagnosis of the disease (24). Indicators of diabetes included an FBG level ≥ 7.0 mmol/L, a 2 h-OGTT level ≥ 11.1 mmol/L, typical diabetes symptoms with a random blood glucose level of 11.1 mmol/L or higher, or a previously reported diagnosis of diabetes (25, 26). Hyperlipidemia was defined as a TC level of 6.2 mmol/L (240 mg/dL) or higher, TG of 2.3 mmol/L (200 mg/dL) or higher, LDL-C of 4.1 mmol/L (160 mg/dL) or higher, HDL-C of less than 1.0 mmol/L (40 mg/dL) (27). Hyperuricemia was defined as a serum uric acid level of 420 mmol/L (7.0 mg/dL) or higher in men and 360 mmol/L (6.0 mg/dL) or higher in women (28, 29). Salt intake was subdivided into mild (<5 g/day), moderate (5–10 g/day), and heavy (>10 g/day) based on self-reported information provided by the study participants. Participants who reported smoking at least one cigarette per day in the past 12 months were categorized as smokers. Body mass index (BMI) was classified as underweight (<18.5 Kg/m^2^), normal (18.5–23.9 Kg/m^2^), overweight (24.0–27.9 Kg/m^2^), and obesity (≥28.0 Kg/m^2^).

### 2.4 Statistical analysis

Absolute and relative frequencies were used to describe demographic characteristics of participants. Variables were presented as proportions and compared using design-adjusted chi-square tests. Using clinical assessment data as the reference, the sensitivity, specificity, under-reporting (1-sensitivity), over-reporting (1-specificity), and agreement for each self-reported chronic condition were also calculated. Sensitivity represents the proportion of participants with a specific chronic condition who accurately self-reported having that condition. Specificity refers to the proportion of participants without a specific chronic condition who accurately self-reported not having that condition. Log-binomial regression models were fitted to examine the potential factors affecting the under-reporting for each chronic condition, with a prevalence ratio (PR) estimated with a 95% confidence interval (CI). Taking into account the clustered nature of the data, we corrected the data estimation error using weighting method based on information from our sampling frame (Supplementary Figure S1) of the survey data. All statistical analysis and graphical presentations were conducted using R software (version 4.2.2). A two-sided *p*-value less than 0.05 was considered statistically significant.

## 3 Results

### 3.1 Characteristics of study participants

A total of 2,272 participants were included in the study, with 1,194 (55.63%) residing in urban areas and 1,168 (51.45%) being females. The majority of participants were young (44.20%), and 41.87% had completed university or higher education. The largest occupational group was farmers (32.20%), with 62.77% of them having an annual family income of RMB 10,000 or lower. Table 1 provides detailed information on the distribution of participants across various characteristics.

**Table 1:** Weighted demographic characteristics of participants.

Variables	Total (*N* = 2,272)
Area (%)	
Urban	1,194 (55.63)
Rural	1,078 (44.37)
Age (%)	
Young (18–39 years)	1,000 (44.20)
Middle (40–59 years)	885 (38.92)
Senior (≥60 years)	387 (16.87)
Sex (%)	
Male	1,104 (48.55)
Female	1,168 (51.45)
Education (%)	
Middle school and below	814 (34.34)
High school	539 (23.79)
College and above	919 (41.87)
Occupation (%)	
Farmer	780 (32.20)
Worker	499 (22.92)
Staff	620 (28.38)
Other	373 (16.50)
Family income (CNY, %)	
Less than 10 thousand	1,467 (62.77)
10 to 50 thousand	529 (24.43)
More than 50 thousand	276 (12.81)
Salt intake (%)	
Mild	427 (18.59)
Moderate	1,616 (71.22)
Heavy	229 (10.29)
Smoke (%)	
No	1,589 (70.33)
Yes	683 (29.67)
Family history of thyroid diseases (%)	
No	2,152 (94.60)
Yes	120 (5.40)
BMI (%)	
Normal	1,144 (50.38)
Underweight	121 (5.32)
Overweight	743 (32.69)
Obesity	264 (11.61)

BMI, body mass index.

### 3.2 Consistency between self-reported and clinical assessment of chronic conditions

Based on the reference of clinical assessment, half of the chronic conditions displayed under-reporting exceeding 50% in self-reports. The highest under-reporting was observed for goiter (85.93, 95% CI: 85.25–86.62%), hyperuricemia (83.94, 95% CI: 83.22–84.66%), and thyroid nodules (72.89, 95% CI: 72.02–73.76%), corresponding to the lowest sensitivity. All chronic conditions exhibited high specificity (greater than 95%), except for hyperlipidemia (67.89, 95% CI: 66.98–68.81%). The over-reporting was the complete opposite, with the top three values being hyperlipidemia (31.11, 95% CI: 31.19–33.02%), hyperuricemia (4.07, 95% CI: 3.69–4.46%), and thyroid nodules (2.32, 95% CI: 2.03–2.62%). Of the eight chronic conditions, hyperlipidemia (62.13, 95% CI: 61.18–63.08%) had the worst concordance between self-report and clinical assessment, followed by hyperuricemia (83.55, 95% CI: 82.82–84.28%) and thyroid nodules (89.81, 95% CI: 89.21–90.40%), while the agreement for the other five conditions was above 90% (Table 2).

**Table 2:** Consistency between self-reported disease diagnosis and clinical assessment.

Diseases	Self-report diagnosis	Clinical assessment		Agreement (95% CI), %	Sensitivity (95% CI), %	Specificity (95% CI), %	Under-reporting (95% CI), %	Over-reporting (95% CI), %
		+	−					
Goiter	+	5	19	97.74 (97.45, 98.03)	14.07 (13.38, 14.75)	99.12 (98.93, 99.30)	85.93 (85.25, 86.62)	0.88 (0.70, 1.07)
	−	31	2,217					
Hyperuricemia	+	54	76	83.55 (82.82, 84.28)	16.06 (15.34, 16.78)	95.93 (95.54, 96.31)	83.94 (83.22, 84.66)	4.07 (3.69, 4.46)
	−	294	1,848					
Thyroid nodules	+	65	45	89.81 (89.21, 90.40)	27.11 (26.24, 27.98)	97.68 (97.38, 97.97)	72.89 (72.02, 73.76)	2.32 (2.03, 2.62)
	−	181	1,981					
Hyperlipidemia	+	359	490	62.13 (61.18, 63.08)	49.84 (48.86, 50.82)	67.89 (66.98, 68.81)	50.16 (49.18, 51.14)	32.11 (31.19, 33.02)
	−	373	1,050					
Diabetes	+	166	0	92.92 (92.41, 93.42)	50.92 (49.94, 51.90)	100.00 (99.82, 100.00)	49.08 (48.10, 50.06)	0.00 (0.00, 0.18)
	−	163	1,943					
Hypertension	+	370	0	90.30 (89.72, 90.88)	62.57 (61.62, 63.52)	100.00 (99.79, 100.00)	37.43 (36.48, 38.38)	0.00 (0.00, 0.21)
	−	222	1,680					
Hypothyroidism	+	33	38	97.57 (97.26, 97.87)	68.43 (67.51, 69.34)	98.23 (97.97, 98.49)	31.57 (30.66, 32.49)	1.77 (1.51, 2.03)
	−	16	2,185					
Hyperthyroidism	+	28	12	99.24 (99.06, 99.41)	84.88 (84.18, 85.58)	99.45 (99.31, 99.60)	15.12 (14.42, 15.82)	0.55 (0.40, 0.69)
	−	5	2,227					

+, positive; −, negative; CI, confidence interval.

### 3.3 Distribution of the inconsistency by patients and diseases

**Figure 2:**
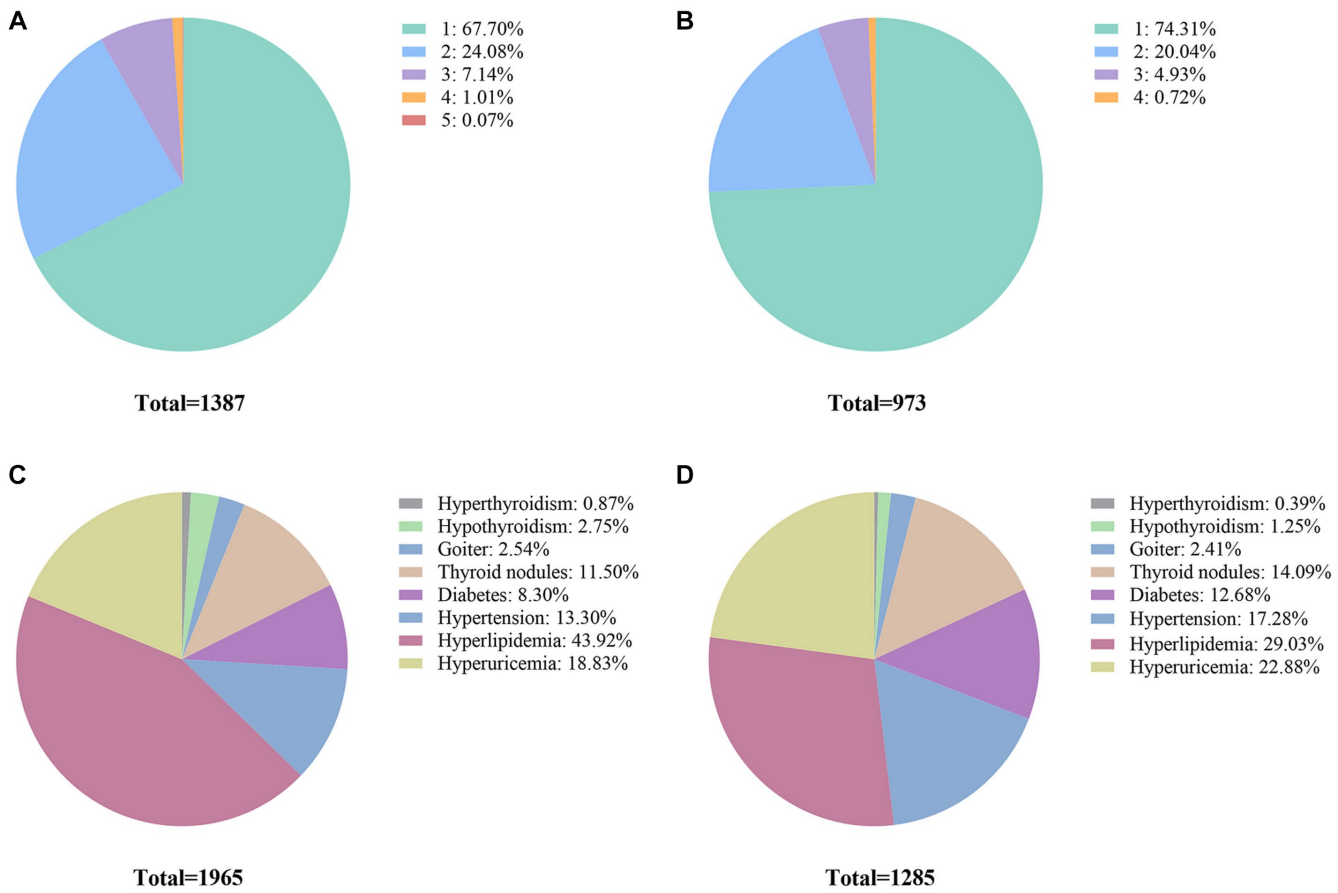
Sample-wise and disease-wise distribution of inconsistency between self-reported disease diagnosis and clinical assessment. **(A)** Proportions of participants for different numbers of diseases (including both under-reporting and over-reporting patients). **(B)** Under-reporting for different numbers of diseases. **(C)** Proportions of participant disease for different types of diseases (including both under-reporting and over-reporting patients). **(D)** Proportions of under-reporting patient diseases for different types of diseases.

Regarding the number of detected diseases, among the surveyees who were inconsistent between self-reported and clinical assessment of the chronic conditions, 67.70% of them were related to one disease (Figure 2A). When considering under-reporting, 74.31% of the study participants failed to correctly report one disease, and 20.04% failed to report two diseases (Figure 2B). In terms of the mistakenly reported disease type, the top three diseases with inconsistency between self-reported and clinical assessment were hyperlipidemia, hyperuricemia, and hypertension, accounting for 43.92, 18.83, and 13.30%, respectively (Figure 2C). Similarly, when only referring to the under-reporting, the distribution pattern of mistakenly reported disease types is similar to the combined findings. The top three diseases with the most under-reporting patients were also hyperlipidemia, hyperuricemia, and hypertension, accounting for 29.03, 22.88, and 17.28%, respectively (Figure 2D).

### 3.4 Factors associated with inconsistency between self-reported disease diagnosis and clinical assessment

**Figure 3:**
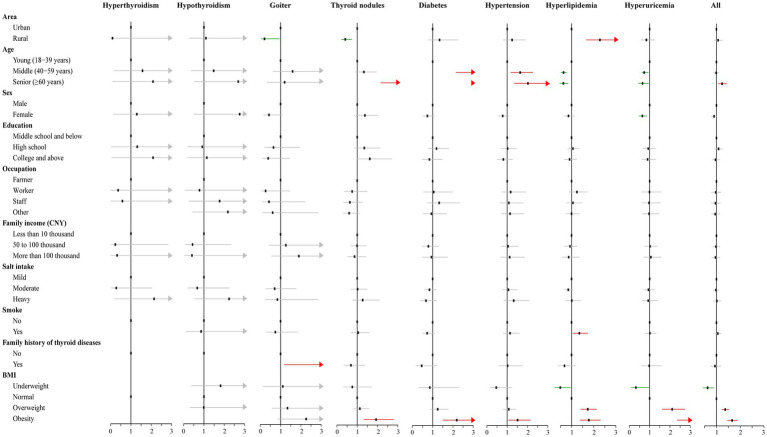
Results of the log-binomial regression for factors associated with under-reporting for the chronic conditions.

The log-binomial regression analysis revealed several factors associated with the under-reporting for each chronic condition (Figure 3). For thyroid disorders, the main influencing factors were area, age, family history of thyroid diseases, and BMI (Supplementary Tables S2–S5). Specifically, rural areas with goiter (PR = 0.21, 95% CI: 0.05–0.93) and thyroid nodules (PR = 0.41, 95% CI: 0.23–0.73) were less likely to under-report the disease status compared to urban areas (*p* < 0.05). Family history of thyroid disease (PR = 3.06, 95% CI: 1.18–7.92) and obesity (PR = 1.93, 95% CI: 1.33–2.79) were associated with increased prevalence of under-reporting of goiter and thyroid nodules. The prevalence of under-reporting increased with increasing BMI for diabetes, hypertension, hyperlipidemia, and hyperuricemia. Older age was also found to be associated with an increased prevalence of under-reporting of diabetes and hypertension, while in contrast, the prevalence of under-reporting of hyperlipidemia and hyperuricemia decreased with age. Additionally, rural area (PR = 2.28, 95% CI: 1.67–3.10) and smoking (PR = 1.35, 95% CI: 1.06–1.73) were identified as potential factors associated with an increased prevalence of under-reporting of hyperlipidemia, while the female sex was a protective factor for under-reporting of hyperuricemia (PR = 0.64, 95% CI: 0.48–0.86), respectively (Supplementary Tables S6–S9). Overall, the findings suggest that senior age and high BMI were potential factors that can affect the correct reporting of chronic disease status in the study population (Supplementary Table S10).

For over-reporting, advanced age emerged as a potential contributor leading participants to inaccurately disclose their true status of hyperlipidemia. Conversely, individuals with a high school diploma and involved in occupations distinct from farming exhibited a higher likelihood of providing accurate and reliable reports regarding their hyperlipidemia condition (Supplementary Table S11).

## 4 Discussion

In the present study, we found that the under-reporting of goiter, hyperuricemia, thyroid nodules, hyperlipidemia, diabetes, hypertension, hypothyroidism, and hyperthyroidism in the study population of Xi’an, China was 85.93, 83.94, 72.89, 50.16, 49.08, 37.43, 31.57, and 15.12%, respectively, which means that in four of these eight conditions, more than half of the patients were not aware of their physical status. When it comes to thyroid nodules, there is no need to be overly concerned about this data. High-resolution ultrasound scans identify thyroid nodules in 19–68% of randomly selected populations, suggesting a possible overdiagnosis of thyroid nodules. This not only leads to the inefficient use of healthcare resources but also places an increasing strain on healthcare services (19, 30). However, for chronic, progressive diseases such as diabetes, a substantial number of under-reported patients can lead to significant public health concerns. As reported by IDF, the number of diabetes patients in China has increased from 90 million to 140 million in the decade from 2011 to 2021, of which about 72.83 million individuals remained undiagnosed, indicating that a staggering 51.7% of the patients were unaware of their condition (4). This is similar to the results of our study, where the under-reporting of diabetes in our study was 49.08%. Meanwhile, data from a national survey revealed that the false-negative rate of self-reported diabetes among middle-aged and older people in China was 58.5% (12). This highlights the challenges in accurately identifying these conditions through self-reporting alone. If disease prevalence is estimated based on self-reported, the prevalence of various diseases will be underestimated to varying degrees. Specifically, the prevalence of goiter, hyperuricemia, thyroid nodules, and hyperlipidemia is underestimated by more than half. It is important to consider various factors, including residential area, age, sex, smoking status, and BMI, that may be associated with the inconsistency between self-reported and clinical assessment of chronic conditions. Previous research has also emphasized the significance of these factors in understanding the consistency of disease reporting (17, 18, 31).

In all eight studied conditions, there was a significant correlation between age and under-reporting for five of the diseases in our study. Similar results regarding the effect of age on the incorrect report of hypertension and diabetes in China have been also described by Xie et al. (7) in 2015, while in another regional study in Guangzhou, researchers observed no association with age (11). Meanwhile, under-reporting of hyperlipidemia and hyperuricemia decreased with age, which was also observed for hyperlipidemia in a previous study in Australia (32). The increase in under-reporting with age can be attributed to several reasons. Firstly, as age increases, the body undergoes common physiological changes and symptoms such as fatigue, joint pain, and memory decline. These symptoms are to some extent considered normal aging processes, leading older individuals to lack sensitivity to potential disease symptoms and often attribute them to natural aging, without actively seeking medical help. Secondly, some diseases may present atypical symptoms in older individuals or symptoms similar to other diseases, making diagnosis more challenging. Older individuals often face the coexistence of multiple chronic conditions, adding complexity to the difficulty of diagnosing and detecting specific diseases. Additionally, with age, some older individuals may experience cognitive decline, such as memory loss and lack of concentration. This can weaken their awareness of their physical condition, reduce their ability to perceive diseases, and sometimes even make it difficult for them to accurately describe their symptoms or seek appropriate medical help. It should be noted that with the promotion of diagnostic and health services and the popularization of health education, people are paying increasing attention to their health. Additionally, common diseases can be diagnosed and treated in primary healthcare institutions, which may partially explain the higher consistency in hyperlipidemia and hyperuricemia.

BMI served as another substantial contributor to the under-reporting of five of the eight diseases. In the current study, we found a positive association between abnormal BMI and a higher prevalence of under-reporting of conditions, whereas no such association was observed in a study conducted in Guangzhou, China (11). Another study carried out in Canada demonstrated a decreasing trend in chronic disease concordance with increasing BMI (31). It was reported that BMI was a consistent determinant of misreporting (33). Obese individuals are more likely to be unaware of their disease status. On one hand, symptoms of certain diseases may be masked or concealed in obese bodies. For example, symptoms of hyperlipidemia and diabetes may be less noticeable in obese individuals, leading to delayed or missed opportunities for early diagnosis and treatment. On the other hand, obese individuals may have biases in self-perception, inaccurately judging their health conditions. They may deny or underestimate their weight and body shape, thereby neglecting the health risks associated with obesity. Additionally, some obese individuals may lack awareness of health issues. They may be unaware of the close association between obesity and a range of diseases such as cardiovascular diseases and endocrine system problems (34, 35). This lack of health consciousness may fail to take action to understand their health status or seek professional medical advice.

In our study, area, sex, smoking, and family history of thyroid diseases were also ascertained as important factors associated with the under-reporting. Rural residents exhibited a higher likelihood of providing accurate reports and were more knowledgeable about their thyroid disorder status, while the opposite was true for hyperlipidemia. Female are more inclined to pay attention to their health status and attach more importance to the consistency of self-reporting diseases (36). In contrast, males may overlook or underestimate certain symptoms, leading to a higher undiagnosed rate of diseases. Smoking was identified as a factor influencing the inconsistency of hypertension reporting, while a family history of thyroid diseases was a potential one for goiter. Smokers exhibited a higher likelihood of providing inaccurate reports regarding their disease status compared to non-smokers. Similarly, individuals with goiter and a family history of thyroid diseases were more susceptible to inaccurately reporting their disease status.

Of the eight chronic conditions, hyperlipidemia had the highest level of over-reporting, with a far greater proportion than the other conditions. On one hand, the over-reporting of hyperlipidemia by some research subjects may be attributed to subjective misperception. Some individuals may erroneously perceive themselves as suffering from hyperlipidemia based on subjective feelings, when, in reality, this may not be the case. On the other hand, misinformation from unreliable sources could also contribute to this phenomenon. Research subjects may have obtained information about hyperlipidemia from inaccurate sources such as the internet or social media, leading to a distorted self-assessment of their condition. Additionally, inaccuracies in the methods used to measure blood lipid levels may also partially account for the high rate of over-reporting among the research subjects.

Given the substantial inconsistency between self-reported diagnosis and clinical assessment, it is imperative to implement potential strategies to address this issue. Firstly, the promotion of health education among the general public is of paramount significance. Providing the public with education on disease symptoms, risk factors, and fostering collaboration with healthcare professionals for accurate self-reporting is crucial. Secondly, the development of more precise self-reporting tools is essential. The utilization of more accurate self-reporting tools, such as structured questionnaires or digital platforms, can aid in enhancing patients’ ability to provide detailed and consistent information about their health status, thereby reducing the inconsistencies in self-reports. Thirdly, the incorporation of objective biomarker measurements can significantly contribute to a comprehensive and accurate assessment of health status when combined with self-reports. Biomarker measurements can serve as a supplement to self-reports, enabling healthcare professionals to gain a better understanding of patients’ health status. Lastly, the promotion of universal access to healthcare services is crucial. Improving the accessibility of healthcare services and encouraging timely medical consultations can mitigate reliance on self-reports and enhance the accuracy of diagnosis and treatments through clinical assessment.

Due to the limited scope of data collection, we were unable to assess the role of other potentially important factors such as drinking status, physical activity, and accessibility of healthcare services on the consistency of self-reported and clinical assessment outcomes of chronic condition, and thus replication in other study populations is needed to assess the impact of these factors. Another limitation is that the current study is based on data collected before the COVID-19 pandemic. Since 2020, due to the impact of COVID-19, there have not been similar large-scale community-based surveys conducted. Therefore, the present study could not provide any knowledge on relevant topics in the post-COVID-19 era. In addition, some indicators such as blood pressure were measured during a single clinical examination, whereas, according to clinical guidelines, diagnosis of hypertension required monitoring of abnormal values at multiple time points. Moreover, the cut-off values such as serum uric acid used for hyperuricemia definition or cholesterol used for hyperlipidemia were not consensual in the literature, which could limit the comparability of our results with other studies. Besides, the study sample is primarily derived from residents living in the vicinity of the Xi’an metropolitan area. The demographic composition of this population may differ from the national average, potentially impacting the representativeness of the research sample to a certain extent. And subject participation rates may similarly affect the comparability of study results. Due to the low frequency of over-reporting errors, it was not possible to investigate the factors associated with over-reporting for the remaining conditions other than hyperlipidemia. Finally, only eight chronic conditions were included in this study, and comprehensive studies including more chronic conditions are still needed in the future.

In conclusion, the self-reported disease diagnoses by respondents and clinical assessment data exhibit significant inconsistency for all eight chronic conditions. Large proportions of patients with multiple chronic conditions were under-reported in Xi’an, China. Combining relevant potential factors, targeted health screenings for high-risk populations might be an effective method for identifying under-reporting patients.

## Data availability statement

The raw data supporting the conclusions of this article will be made available by the authors, without undue reservation.

## Ethics statement

The studies involving humans were approved by Medical Ethics Committee of The First Affiliated Hospital of China Medical University. The studies were conducted in accordance with the local legislation and institutional requirements. The participants provided their written informed consent to participate in this study.

## Author contributions

HL: Conceptualization, Data curation, Formal analysis, Visualization, Writing – original draft, Writing – review & editing. YZ: Data curation, Investigation, Writing – review & editing. LQ: Data curation, Formal analysis, Investigation, Writing – review & editing. CY: Investigation, Methodology, Visualization, Writing – review & editing. YY: Investigation, Methodology, Visualization, Writing – review & editing. TZ: Conceptualization, Supervision, Writing – original draft, Writing – review & editing. QW: Conceptualization, Supervision, Writing – review & editing. JH: Writing – review & editing.
