# Supplementary material for: Consistency between self-reported disease diagnosis and clinical assessment and under-reporting for chronic conditions: data from a community-based study in Xi’an, China
Source: Front Public Health. 2024 Jan 16;12:1296939. doi: 10.3389/fpubh.2024.1296939 (PMC10825002; doi:10.3389/fpubh.2024.1296939)
Supplement: Supplementary file 1 [file Data_Sheet_1.PDF]

Supplementary Material

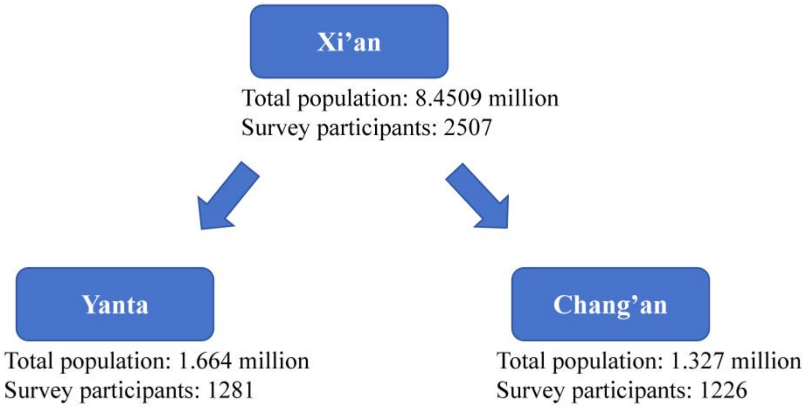

Figure S1. Sampling frame of the survey data collected in Xi'an, 2017.

Table S1. Diagnostic criteria for thyroid disorders.

**Thyroid disorders**	**Diagnostic criteria**
Hyperthyroidism	TSH < 0.27 mIU/L, FT4 > 22.00 pmol/L, or FT3 > 6.80 pmol/L
Hypothyroidism	TSH > 4.20 mIU/L, FT4 < 12.00 pmol/L
Goiter	Thyroid volume > 22.5 mL (men) or > 25.4 mL (women)
Thyroid nodules	One or more nodules (> 5 mm) without goiter

TSH, thyroid stimulating hormone; FT4, free thyroxine; FT3, free triiodothyronine.

Table S2. Log-binomial analysis for under-reported patients of hyperthyroidism.

Variables	PR (95% CI)	*P* value
**Area**		
Rural	Reference	
Urban	0.09 (0.00-5.14)	0.245
**Age**		
Young (18-39 years)	Reference	
Middle (40-59 years)	1.57 (0.17-14.67)	0.695
Senior (≥60 years)	2.09 (0.10-42.41)	0.632
**Sex**		
Male	Reference	
Female	1.29 (0.13-12.69)	0.826
**Education**		
Middle school and below	Reference	
High school	1.31 (0.04-40.74)	0.878
College and above	2.09 (0.06-74.64)	0.685
**Occupation**		
Farmer	Reference	
Worker	0.37 (0.00-35.44)	0.669
Staff	0.58 (0.01-40.21)	0.799
Other		
**Family income (CNY)**		
Less than 10 thousand	Reference	
50 to 100 thousand	0.23 (0.02-2.83)	0.253
More than 100 thousand	0.32 (0.02-4.80)	0.411
**Salt intake**		
Mild	Reference	
Moderate	0.28 (0.04-2.03)	0.206
Heavy	2.14 (0.19-24.52)	0.542
**Smoke**		
No	Reference	
Yes	-	-
**Family history of thyroid diseases**		
No	Reference	
Yes	-	-
**BMI**		
Underweight	-	-
Normal	Reference	
Overweight	-	-
Obesity	-	-

PR, prevalence ratio; CI, confidence interval; BMI, body mass index; -, without value.

Table S3. Log-binomial analysis for under-reported patients of hypothyroidism.

Variables	PR (95% CI)	*P* value
**Area**		
Rural	Reference	
Urban	1.10 (0.26-4.71)	0.901
**Age**		
Young (18-39 years)	Reference	
Middle (40-59 years)	1.48 (0.36-6.12)	0.590
Senior (≥60 years)	2.69 (0.52-13.81)	0.235
**Sex**		
Male	Reference	
Female	2.76 (0.50-15.13)	0.243
**Education**		
Middle school and below	Reference	
High school	0.92 (0.20-4.36)	0.920
College and above	1.14 (0.20-6.38)	0.882
**Occupation**		
Worker	Reference	
Farmer	0.78 (0.06-10.16)	0.846
Staff	1.77 (0.26-11.96)	0.560
Other	2.18 (0.44-10.69)	0.338
**Family income (CNY)**		
Less than 10 thousand	Reference	
50 to 100 thousand	0.44 (0.08-2.35)	0.338
More than 100 thousand	0.41 (0.04-4.06)	0.443
**Salt intake**		
Mild	Reference	
Moderate	0.67 (0.20-2.25)	0.517
Heavy	2.24 (0.53-9.55)	0.275
**Smoke**		
No	Reference	
Yes	0.86 (0.12-6.27)	0.884
**Family history of thyroid diseases**		
No	Reference	
Yes	-	-
**BMI**		
Underweight	1.82 (0.39-8.45)	0.443
Normal	Reference	
Overweight	0.99 (0.32-3.03)	0.985
Obesity	-	-

PR, prevalence ratio; CI, confidence interval; BMI, body mass index; -, without value.

Table S4. Log-binomial analysis for under-reported patients of goiter.

Variables	PR (95% CI)	*P* value
**Area**		
Rural	Reference	
Urban	0.21 (0.05-0.93)	0.040
**Age**		
Young (18-39 years)	Reference	
Middle (40-59 years)	1.59 (0.64-3.98)	0.318
Senior (≥60 years)	1.20 (0.34-4.17)	0.776
**Sex**		
Male	Reference	
Female	0.43 (0.17-1.06)	0.065
**Education**		
Middle school and below	Reference	
High school	0.65 (0.22-1.95)	0.446
College and above	0.39 (0.10-1.45)	0.159
**Occupation**		
Worker	Reference	
Farmer	0.26 (0.05-1.46)	0.126
Staff	0.43 (0.08-2.20)	0.309
Other	0.61 (0.13-2.83)	0.523
**Family income (CNY)**		
Less than 10 thousand	Reference	
50 to 100 thousand	1.26 (0.44-3.65)	0.667
More than 100 thousand	1.90 (0.56-6.43)	0.302
**Salt intake**		
Mild	Reference	
Moderate	0.71 (0.28-1.76)	0.455
Heavy	0.84 (0.25-2.83)	0.783
**Smoke**		
No	Reference	
Yes	0.74 (0.30-1.84)	0.522
**Family history of thyroid diseases**		
No	Reference	
Yes	3.06 (1.18-7.92)	0.021
**BMI**		
Underweight	1.11 (0.14-8.61)	0.923
Normal	Reference	
Overweight	1.34 (0.58-3.10)	0.492
Obesity	2.26 (0.87-5.87)	0.094

PR, prevalence ratio; CI, confidence interval; BMI, body mass index.

Table S5. Log-binomial analysis for under-reported patients of thyroid nodules.

Variables	PR (95% CI)	*P* value
**Area**		
Rural	Reference	
Urban	0.41 (0.23-0.73)	0.002
**Age**		
Young (18-39 years)	Reference	
Middle (40-59 years)	1.33 (0.91-1.94)	0.140
Senior (≥60 years)	3.39 (2.16-5.31)	<0.001
**Sex**		
Male	Reference	
Female	1.38 (0.95-2.01)	0.090
**Education**		
Middle school and below	Reference	
High school	1.35 (0.86-2.12)	0.188
College and above	1.62 (0.97-2.70)	0.065
**Occupation**		
Worker	Reference	
Farmer	0.75 (0.38-1.49)	0.410
Staff	0.64 (0.33-1.25)	0.191
Other	0.60 (0.32-1.13)	0.115
**Family income (CNY)**		
Less than 10 thousand	Reference	
50 to 100 thousand	0.99 (0.68-1.43)	0.937
More than 100 thousand	0.87 (0.53-1.43)	0.573
**Salt intake**		
Mild	Reference	
Moderate	1.02 (0.70-1.48)	0.928
Heavy	1.27 (0.77-2.09)	0.349
**Smoke**		
No	Reference	
Yes	1.05 (0.70-1.59)	0.799
**Family history of thyroid diseases**		
No	Reference	
Yes	0.69 (0.35-1.36)	0.283
**BMI**		
Underweight	0.76 (0.34-1.69)	0.496
Normal	Reference	
Overweight	1.13 (0.82-1.58)	0.451
Obesity	1.93 (1.33-2.79)	<0.001

PR, prevalence ratio; CI, confidence interval; BMI, body mass index.

Table S6. Log-binomial analysis for under-reported patients of diabetes.

Variables	PR (95% CI)	*P* value
**Area**		
Rural	Reference	
Urban	1.34 (0.80-2.26)	0.272
**Age**		
Young (18-39 years)	Reference	
Middle (40-59 years)	3.51 (2.15-5.73)	<0.001
Senior (≥60 years)	6.38 (3.71-10.95)	<0.001
**Sex**		
Male	Reference	
Female	0.74 (0.52-1.06)	0.105
**Education**		
Middle school and below	Reference	
High school	1.19 (0.78-1.79)	0.419
College and above	0.84 (0.48-1.47)	0.547
**Occupation**		
Worker	Reference	
Farmer	1.05 (0.56-1.97)	0.869
Staff	1.32 (0.75-2.33)	0.333
Other	0.94 (0.53-1.67)	0.845
**Family income (CNY)**		
Less than 10 thousand	Reference	
50 to 100 thousand	0.79 (0.48-1.31)	0.364
More than 100 thousand	0.94 (0.50-1.73)	0.832
**Salt intake**		
Mild	Reference	
Moderate	0.84 (0.60-1.19)	0.333
Heavy	0.67 (0.38-1.19)	0.169
**Smoke**		
No	Reference	
Yes	0.73 (0.50-1.08)	0.112
**Family history of thyroid diseases**		
No	Reference	
Yes	0.46 (0.18-1.21)	0.117
**BMI**		
Underweight	0.86 (0.32-2.30)	0.765
Normal	Reference	
Overweight	1.25 (0.89-1.76)	0.189
Obesity	2.19 (1.50-3.21)	<0.001

PR, prevalence ratio; CI, confidence interval; BMI, body mass index.

Table S7. Log-binomial analysis for under-reported patients of hypertension.

Variables	PR (95% CI)	*P* value
**Area**		
Rural	Reference	
Urban	1.24 (0.81-1.90)	0.322
**Age**		
Young (18-39 years)	Reference	
Middle (40-59 years)	1.64 (1.18-2.26)	0.003
Senior (≥60 years)	2.02 (1.35-3.02)	0.001
**Sex**		
Male	Reference	
Female	0.78 (0.56-1.09)	0.147
**Education**		
Middle school and below	Reference	
High school	1.02 (0.71-1.45)	0.934
College and above	0.81 (0.51-1.26)	0.347
**Occupation**		
Worker	Reference	
Farmer	1.17 (0.71-1.92)	0.550
Staff	1.08 (0.65-1.78)	0.768
Other	1.14 (0.72-1.81)	0.575
**Family income (CNY)**		
Less than 10 thousand	Reference	
50 to 100 thousand	1.04 (0.70-1.54)	0.845
More than 100 thousand	1.12 (0.68-1.86)	0.651
**Salt intake**		
Mild	Reference	
Moderate	1.06 (0.76-1.49)	0.733
Heavy	1.32 (0.84-2.07)	0.222
**Smoke**		
No	Reference	
Yes	1.14 (0.82-1.60)	0.430
**Family history of thyroid diseases**		
No	Reference	
Yes	1.02 (0.59-1.76)	0.949
**BMI**		
Underweight	0.46 (0.17-1.22)	0.120
Normal	Reference	
Overweight	1.08 (0.81-1.44)	0.582
Obesity	1.51 (1.07-2.14)	0.020

PR, prevalence ratio; CI, confidence interval; BMI, body mass index.

Table S8. Log-binomial analysis for under-reported patients of hyperlipidemia.

Variables	PR (95% CI)	*P* value
**Area**		
Rural	Reference	
Urban	2.28 (1.67-3.10)	<0.001
**Age**		
Young (18-39 years)	Reference	
Middle (40-59 years)	0.64 (0.52-0.78)	<0.001
Senior (≥60 years)	0.63 (0.47-0.84)	0.002
**Sex**		
Male	Reference	
Female	0.86 (0.66-1.12)	0.266
**Education**		
Middle school and below	Reference	
High school	1.06 (0.83-1.34)	0.649
College and above	0.91 (0.68-1.22)	0.542
**Occupation**		
Worker	Reference	
Farmer	1.24 (0.89-1.71)	0.197
Staff	1.06 (0.77-1.47)	0.707
Other	1.00 (0.74-1.34)	0.974
**Family income (CNY)**		
Less than 10 thousand	Reference	
50 to 100 thousand	0.93 (0.68-1.26)	0.626
More than 100 thousand	0.87 (0.57-1.34)	0.530
**Salt intake**		
Mild	Reference	
Moderate	0.85 (0.69-1.06)	0.150
Heavy	1.01 (0.73-1.40)	0.948
**Smoke**		
No	Reference	
Yes	1.35 (1.06-1.73)	0.016
**Family history of thyroid diseases**		
No	Reference	
Yes	0.68 (0.39-1.18)	0.169
**BMI**		
Underweight	0.49 (0.25-0.96)	0.038
Normal	Reference	
Overweight	1.73 (1.42-2.13)	<0.001
Obesity	1.78 (1.38-2.30)	<0.001

PR, prevalence ratio; CI, confidence interval; BMI, body mass index.

Table S9. Log-binomial analysis for under-reported patients of hyperuricemia.

Variables	PR (95% CI)	*P* value
**Area**		
Rural	Reference	
Urban	0.84 (0.57-1.23)	0.369
**Age**		
Young (18-39 years)	Reference	
Middle (40-59 years)	0.73 (0.57-0.93)	0.009
Senior (≥60 years)	0.65 (0.45-0.95)	0.024
**Sex**		
Male	Reference	
Female	0.64 (0.48-0.86)	0.002
**Education**		
Middle school and below	Reference	
High school	0.93 (0.67-1.29)	0.667
College and above	0.90 (0.62-1.29)	0.567
**Occupation**		
Worker	Reference	
Farmer	0.99 (0.62-1.56)	0.956
Staff	0.97 (0.62-1.53)	0.897
Other	0.97 (0.64-1.46)	0.875
**Family income (CNY)**		
Less than 10 thousand	Reference	
50 to 100 thousand	1.02 (0.76-1.38)	0.886
More than 100 thousand	1.06 (0.72-1.56)	0.764
**Salt intake**		
Mild	Reference	
Moderate	0.94 (0.71-1.24)	0.654
Heavy	0.93 (0.62-1.40)	0.725
**Smoke**		
No	Reference	
Yes	1.01 (0.78-1.31)	0.940
**Family history of thyroid diseases**		
No	Reference	
Yes	0.98 (0.61-1.57)	0.931
**BMI**		
Underweight	0.32 (0.10-0.99)	0.048
Normal	Reference	
Overweight	2.11 (1.62-2.73)	<0.001
Obesity	3.14 (2.36-4.19)	<0.001

PR, prevalence ratio; CI, confidence interval; BMI, body mass index.

Table S10. Log-binomial analysis for under-reported patients of self-reported diseases.

Variables	PR (95% CI)	*P* value
**Area**		
Rural	Reference	
Urban	1.07 (0.91-1.26)	0.430
**Age**		
Young (18-39 years)	Reference	
Middle (40-59 years)	0.99 (0.88-1.11)	0.801
Senior (≥60 years)	1.24 (1.08-1.43)	0.002
**Sex**		
Male	Reference	
Female	0.90 (0.79-1.02)	0.087
**Education**		
Middle school and below	Reference	
High school	1.09 (0.94-1.25)	0.249
College and above	0.96 (0.82-1.14)	0.668
**Occupation**		
Worker	Reference	
Farmer	0.97 (0.79-1.19)	0.782
Staff	0.96 (0.79-1.16)	0.655
Other	0.95 (0.80-1.14)	0.602
**Family income (CNY)**		
Less than 10 thousand	Reference	
50 to 100 thousand	0.98 (0.85-1.13)	0.745
More than 100 thousand	0.96 (0.80-1.15)	0.674
**Salt intake**		
Mild	Reference	
Moderate	0.98 (0.87-1.10)	0.723
Heavy	1.02 (0.87-1.20)	0.785
**Smoke**		
No	Reference	
Yes	1.06 (0.94-1.20)	0.365
**Family history of thyroid diseases**		
No	Reference	
Yes	0.94 (0.76-1.18)	0.611
**BMI**		
Underweight	0.63 (0.44-0.89)	0.009
Normal	Reference	
Overweight	1.37 (1.23-1.53)	<0.001
Obesity	1.66 (1.47-1.88)	<0.001

PR, prevalence ratio; CI, confidence interval; BMI, body mass index.

Table S11. Log-binomial analysis for over-reported patients of hyperlipidemia.

Variables	PR (95% CI)	*P* value
**Area**		
Rural	Reference	
Urban	0.37 (0.28-0.48)	<0.001
**Age**		
Young (18-39 years)	Reference	
Middle (40-59 years)	1.85 (1.50-2.28)	<0.001
Senior (≥60 years)	2.02 (1.54-2.66)	<0.001
**Sex**		
Male	Reference	
Female	1.00 (0.82-1.22)	0.988
**Education**		
Middle school and below	Reference	
High school	0.75 (0.59-0.96)	0.021
College and above	0.79 (0.60-1.04)	0.096
**Occupation**		
Worker	Reference	
Farmer	0.66 (0.48-0.92)	0.014
Staff	0.70 (0.50-0.96)	0.030
Other	0.57 (0.42-0.79)	0.001
**Family income (CNY)**		
Less than 10 thousand	Reference	
50 to 100 thousand	0.90 (0.72-1.11)	0.328
More than 100 thousand	0.86 (0.65-1.13)	0.274
**Salt intake**		
Mild	Reference	
Moderate	0.96 (0.79-1.18)	0.716
Heavy	0.86 (0.64-1.16)	0.319
**Smoke**		
No	Reference	
Yes	1.05 (0.85-1.30)	0.674
**Family history of thyroid diseases**		
No	Reference	
Yes	1.32 (1.00-1.74)	0.054
**BMI**		
Underweight	0.77 (0.49-1.22)	0.265
Normal	Reference	
Overweight	1.05 (0.89-1.24)	0.573
Obesity	0.91 (0.70-1.18)	0.474

PR, prevalence ratio; CI, confidence interval; BMI, body mass index.
